# Supplementary material for: Complex‐centric proteome profiling by SEC‐SWATH‐MS
Source: Mol Syst Biol. 2019 Jan 14;15(1):e8438. doi: 10.15252/msb.20188438 (PMC6346213; doi:10.15252/msb.20188438)
Supplement: Supplementary file 8 — Dataset EV7 [file MSB-15-e8438-s008.zip › feature_plots_string/Q7KZN9.pdf]

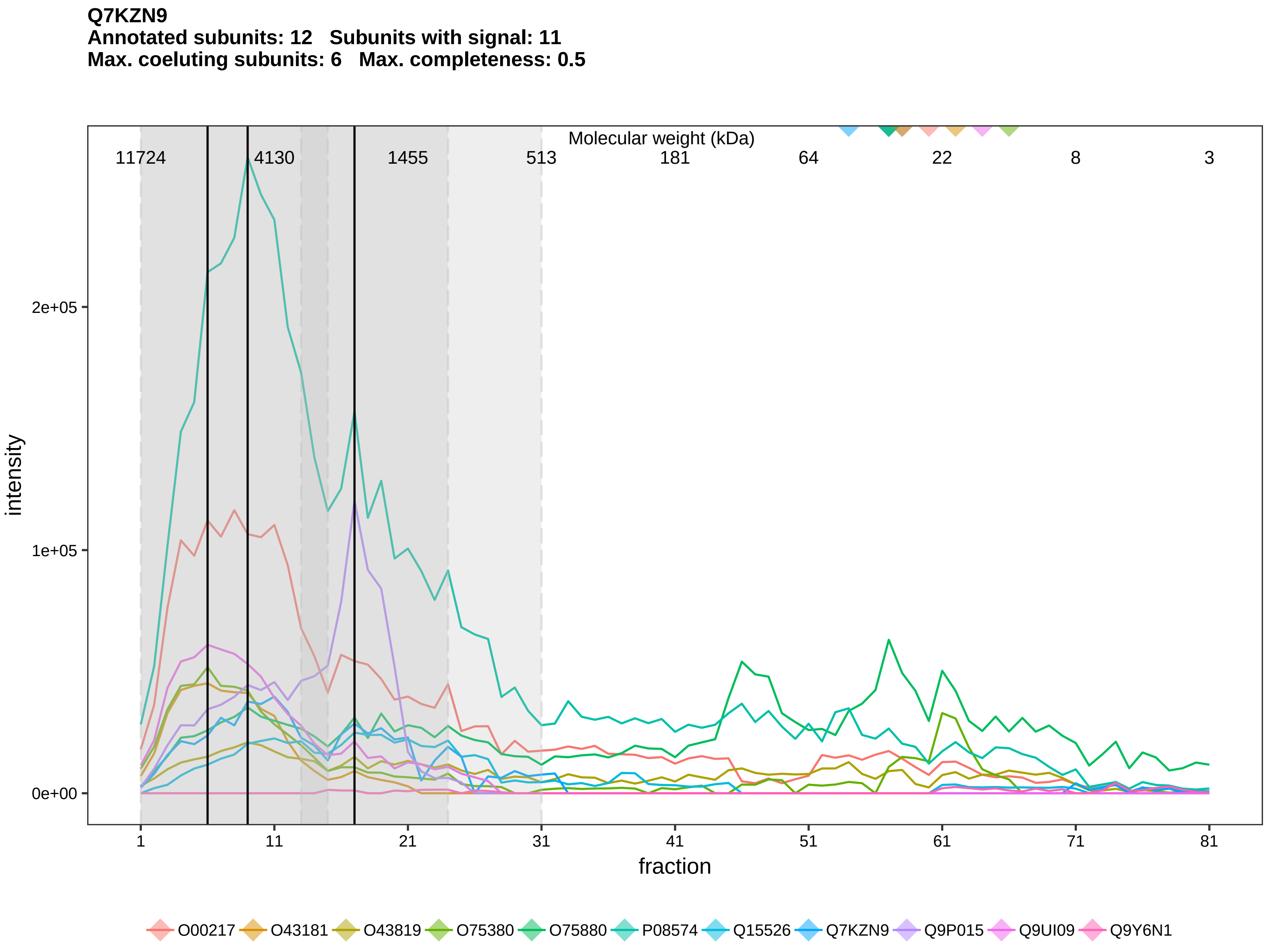
Q7KZN9
Annotated subunits: 12 Subunits with signal: 11
Max. coeluting subunits: 6 Max. completeness: 0.5
Molecular weight (kDa)
11724
4130
1455
513
181
64
22
8
3
intensity
2e+05
1e+05
0e+00
1
11
21
31
41
51
61
71
81
fraction
O00217
O43181
O43819
O75380
O75880
P08574
Q15526
Q7KZN9
Q9P015
Q9UI09
Q9Y6N1
